# Anticonvulsant role of nitric oxide synthase inhibitors in epilepsy (Review)

**DOI:** 10.3892/mi.2026.340

**Published:** 2026-09-15

**Authors:** Ercan Ozdemir

**Affiliations:** Department of Physiology, Faculty of Medicine, Sivas Cumhuriyet University, 58140 Sivas, Turkey

**Keywords:** epilepsy, nitric oxide, nitric oxide synthase inhibitors, anticonvulsants, excitotoxicity

## Abstract

Epilepsy is a pathophysiological process triggered by an imbalance between excitatory and inhibitory mechanisms in the brain, resulting in the sudden and synchronized excessive electrical discharges of neurons. Nitric oxide (NO) is a cellular signaling molecule that functions as both a neurotransmitter and neuromodulator in the central nervous system. Despite its critical role in neuronal function, the impact of the NO pathway on the pathophysiology of epilepsy remains complex. NO, produced by different isoforms of the NO synthase (NOS) enzyme, exerts a bidirectional effect on epileptogenesis. In the present review, the effects of non-selective NOS inhibitors (L-NAME) and isoform-specific selective neuronal NOS (nNOS; 7-nitroindazole) and inducible NOS (iNOS; aminoguanidine) inhibitors on seizure threshold, seizure duration and neuroinflammation are comparatively discussed. Evidence indicates that the selective inhibition of nNOS and iNOS activity significantly suppresses glutamatergic hyperexcitability and nitrosative stress associated with N-methyl-D-aspartate receptor activation. nNOS inhibition increases neuronal survival and reduces seizure severity by decreasing intracellular Ca^2+^ influx. Conversely, endothelial NOS inhibition has been shown to produce proconvulsant effects by reducing cerebral perfusion. This situation highlights the critical importance of isoform specificity in anticonvulsant efficacy. Unlike classical antiepileptic drugs, NOS inhibitors stand out as potent anticonvulsant agents by targeting cellular signaling and inflammation pathways, rather than directly targeting ion channels. Current findings suggest that nNOS- and iNOS-targeted strategies may be strong therapeutic candidates for next-generation combination therapies, particularly in cases of refractory epilepsy. The present review summarizes the roles of the NO signaling pathway in seizure mechanisms and discusses the anticonvulsant potential of NOS inhibitors in the treatment of epilepsy.

## 1. Introduction

Epilepsy is a complex neurological disorder characterized by abnormal synchronized neural activity in the brain, resulting in memory impairment, loss of consciousness, and sensory disturbances. It has a prevalence of 0.3-0.5% worldwide, and its incidence is estimated to be between 5 and 10 per 1,000 individuals in different countries (1,2). Due to the lack of a complete understanding of the pathophysiology of the disease, research into novel drugs for the treatment of epilepsy remains ongoing (3). Furthermore, although numerous antiepileptic drugs are currently used to control the disease, drug resistance developing in approximately one-third of patients and severe neurological side-effects that reduce the quality of life of patients limit the clinical success of current treatments. These shortcomings necessitate the development of novel, more effective and reliable therapeutic strategies. The disruption of the balance between excitatory and inhibitory neurotransmitters triggers cellular oxidative stress; while increased reactive oxygen species (ROS) increase nitric oxide (NO) production and consequent peroxynitrite formation, creating a vicious cycle that accelerates neurodegeneration (4,5). Furthermore, NO, a retrograde messenger, is a potential neurotransmitter linked to the regulation of brain excitability and the induction of seizure activity (6).

NO is synthesized from L-arginine and plays critical roles in various neuroinflammatory disorders due to its prooxidant properties (7). The activation of soluble guanylyl cyclase induces the formation of cyclic guanosine monophosphate (cGMP) (6). The activation of the glutamate and N-methyl-D-aspartate (NMDA) receptor leads to an increase in Ca^2+^ in brain cells, resulting in the activation of neuronal NO synthase (nNOS) (8). Activated nNOS enables the synthesis of NO from L-arginine. nNOS inhibition reduces the frequency of epileptic seizures in epileptic animals by preventing neuronal damage (9). Although NO has antioxidant properties, in some cases, it can play crucial roles in neurodegenerative disorders due to its prooxidant effects (10,11). The increased production of cGMP in neurons stimulates NMDA receptor (NMDAR) (12). Consequently, Ca^2+-^calmodulin-dependent systems are activated, thus continuously inducing the overproduction of NO with nNOS (6). During epileptic seizures, excessive NO production inactivates mitochondrial respiratory enzymes and results in impaired oxidative phosphorylation (13). Mitochondrial respiratory chain enzymes are not resistant to excessive amounts of NO and peroxynitrite products. Therefore, excess NO leads to impaired mitochondrial respiration function. Impaired mitochondrial function in neuronal cells leads to apoptosis and ultimately cellular death (14,15).

Previous studies have focused on the differing and controversial roles of NO in the central and peripheral nervous systems (16,17). Some studies have shown that NO exerts anticonvulsant (18,19) and proconvulsant effects (20). It has been reported that the controversial results in epilepsy models may stem from the inappropriate use of NOS inhibitors in terms of dose and the timing of treatment, method of seizure induction, solvents used as carriers and the route of administration (16).

NO, produced by glial cells in the central nervous system, has been reported to exert cytotoxic effects (21). Therefore, agents that block NO production may reduce neural excitability and improve brain function through their neuroprotective effects. The present review aimed to discuss the potential therapeutic effects of neuronal NO-cGMP pathway inhibitors in epileptic seizures.

## 2. Pathophysiology of epilepsy

Under normal physiological conditions, the electrical activity of the brain is not synchronized. By contrast, in epilepsy, the electrical activity of the brain consists of synchronized neuronal discharges. These abnormal synchronized discharges are associated with a specific group of neurons in the cerebral cortex known as the epileptic focus (22). Subsequently, this focus tends to spread to other areas of the brain, resulting in symptoms, such as abnormal sensations and thoughts (23). In general, epileptic seizures result from the abnormal transmission of neuronal impulses and occur due to the overstimulation of neurons. Under physiological conditions, excitatory neurons do not develop a new action potential due to hyperpolarization that develops over time. By contrast, during seizures, there is a decrease in the neuronal excitability threshold, which increases neuronal excitability (24-26).

Antiepileptic drugs exert their effects primarily through three key mechanisms: The inactivation of ion channels (such as sodium), increased inhibitory gamma-aminobutyric acid-dergic (GABAergic) transmission, and the inhibition of excitatory glutamatergic transmission (27,28). Voltage-dependent sodium/calcium channels located in the neuron membrane open with excitatory signals, leading to rapid depolarization. This, in turn, causes the release of certain neurotransmitters at the axon terminal. Some antiepileptic drugs function by blocking these channels and have high selectivity for abnormal depolarizations (29).

Of note, some antiepileptic drugs reduce excitability by creating hyperpolarization in neurons through the activation of potassium channels (30,31). Antiepileptic drugs that function via GABA receptors either increase the release of the GABA inhibitory neurotransmitter or activate chloride channels coupled to GABAA receptors (32,33). One of the causes of epilepsy is the overactivation of NMDA receptors by glutamate. Therefore, some antiepileptic drugs exert their effects through the inhibition of glutamatergic stimulation (34,35). Drugs that function via glutamate receptors include those targeting α-amino-3-hydroxy-5-methyl-4-isoxazolepropionic acid receptors and the presynaptic release mechanism of synaptic vesicle protein 2A and the α2δ subunit of voltage-gated calcium channels (36).

## 3. Epileptogenesis

Epileptogenesis is the process by which functional changes occur in a previously normally functioning brain network, leading to increased seizure susceptibility and, consequently, to an increased likelihood of seizures (37-39). Furthermore, there is evidence to indicate that the frequency of spontaneous seizures continues to increase after the initial spontaneous seizure (40-42). This indicates that epileptogenesis is not an acute process, but a continuous and long-term one. Various molecular and cellular changes that trigger the first seizure then continue persistently, leading to the progression of epilepsy (37,43).

The vast majority of information underlying the molecular mechanisms of epileptogenesis has been derived from studies focusing on the hippocampus. This information demonstrates that excitatory positive feedback is a necessary feature of epileptic networks (44,45). Synaptic connections can switch back and forth between normal and epileptic modes of activity and exhibit bivariate stability (46). Of note, two possible mechanisms are proposed for the development of positive feedback in the formation of epileptogenesis: The first mechanism is the elimination of inhibitory circuits through the degeneration of mossy cells that excite inhibitory basket cell interneurons in the dentate gyrus (37). Normal hippocampal neural networks have a moderate level of positive feedback due to recurrent glutamatergic connections with major neurons. It has been demonstrated that the proportion of neuron pairs with excitatory interneurons in hippocampal preparations is ~1-3% (47). A second proposed mechanism is the re-sprouting of numerous new synaptic connections among neurons that survive any brain injury (48). Axon sprouting is a consequence of a natural reparative response observed in neurons in various brain injuries (49). The predominance of glutamatergic sprouting in interneurons may lead to epileptogenesis (50). Conversely, the development of molecules that promote the sprouting of GABAergic terminals may suppress epileptogenesis.

Studies aimed at elucidating the molecular mechanisms of epileptogenesis have allowed for the identification of affected molecules, functional gene and protein networks. These include TGF-β and IGF-1 signaling (51), complement activation (52), and gene expression modulation related to glial oxidative stress (53) and mTOR (54).

## 4. Reactive oxygen and nitrogen species in epileptogenesis

During epileptogenesis, seizures lead to persistent mitochondrial dysfunction and an increase in free radicals and pro-inflammatory cytokines in the brain. Physiologically, normal levels of free radical increase are essential for the function of brain cells (55). However, highly increased levels of ROS and peroxynitrite derived from NO lead to the formation of reactive nitrogen species, a toxic compound for molecules such as lipids, proteins and DNA (56,57). Reactive species consist of hydroxyl radicals (OH^-^), superoxide (O_2_^−^) and hydrogen peroxide (H_2_O_2_) molecules. These compounds are synthesized by NOS, NADPH oxidase (NOX) and halo-peroxidases (58). Under physiological conditions, the level of oxidative stress is precisely regulated via the transcription factor, nuclear factor erythroid 2-associated factor 2, and antioxidant response elements (59).

Endogenous antioxidants include enzymes, such as catalase (CAT), glutathione peroxidase (GPx), superoxide dismutase (SOD) and glutathione reductase. In addition, the body contains compounds with antioxidant properties that are not enzymes, such as transferrin, albumin, ferritin, vitamins C and E, and melatonin (17). Although the central nervous system constitutes only 2% of body weight, it consumes a high amount of O_2_, ~20%. Therefore, it exhibits a very high sensitivity to oxidative stress (60). Electron transport chain regions in mitochondria, particularly during ATP synthesis, cause high levels of ROS formation. Furthermore, xanthine oxidase, NOX and cytochrome P450 are other sources of O_2_^−^ (61). Superoxide produced in the body is converted to hydrogen peroxide (H_2_O_2_) by the SOD enzyme under physiological conditions. This is then treated with CAT and GPx to form water (H_2_O) molecules. During epileptogenesis, mitochondrial stress increases, creating an imbalance between oxidants and antioxidant enzyme defenses. In this situation, excess superoxide reacts with H_2_O_2_ to form highly toxic hydroxyl radicals, causing significant damage to brain cells and neurodegeneration (62,63).

NO is synthesized by NOS from arginine, one of the essential amino acids, and NOS has three main isoforms: nNOS, inducible NOS (iNOS) and endothelial NOS (eNOS) (10). Under physiological conditions, NO is produced in small amounts in the brain by eNOS and nNOS, and the NO synthesized in this manner exhibits tissue-protective properties. By contrast, iNOS produces high levels of NO in pathological conditions, particularly in brain injuries and in response to various cytokines, and the NO synthesized by iNOS causes neuronal toxicity (64-66). However, NO synthesized by eNOS prevents atherosclerosis, reduces arterial blood pressure by relaxing smooth muscle and stimulates angiogenesis (67). The activation of NMDAR in neuronal synaptic junctions increases NO synthesis by nNOS. NO activates soluble guanylate cyclase, strengthening cGMP signaling and modulating learning and memory mechanisms (68). There is evidence to suggest that nNOS plays critical roles in kainic acid-induced seizures (69). Increased levels of oxidative stress in the brain lead to excessive superoxide and NO accumulation. These products combine to produce toxic peroxynitrite and dinitrogen trioxide, ultimately resulting in the development of pathologies, such as epileptic seizures and Alzheimer's disease (70).

## 5. NO and epilepsy

NO is a gas molecule that can diffuse freely in tissues and cannot be stored in cells, and it exerts its effects through the activation of soluble guanylate cyclase or the nitrosylation of intracellular proteins. Specifically, S-nitrosylation plays a role in regulating NMDAR activity and blocks excessive Ca^2+^ influx to protect neurons (71,72). It has been demonstrated that the intraperitoneal administration of allopurinol at a dose of 50 mg/kg in mice exerts significant anticonvulsant activity in pentylenetetrazole (PTZ)-induced seizures; pre-treatment with L-arginine enhances the anticonvulsant effects of allopurinol in PTZ-induced seizures (73). It has been suggested that synapic acid exerts anticonvulsant effects by reducing prefrontal cortex nitrite levels and regulating glutamatergic signaling (74).

Whether NO is a neuroprotective or toxic molecule depends on the source of NO production following an injury and the relevant NO synthase isoform (75). There is evidence to indicate differences in the pathophysiological roles of NO in the peripheral and central nervous systems (16,76). The reason why NO exerts anticonvulsant effects in some studies and proconvulsant effects in others is attributed to the types of chemical convulsants used in the studies (12,16). Under normal conditions, NO is synthesized by NADPH-dependent NOS of L-arginine (77). By contrast, in pathological conditions, the activation of NMDARs leads to the increased intracellular expression of Ca^2+^, resulting in high levels of NO production by stimulating Ca²/calmodulin-dependent NOS (78). Furthermore, NMDARs and the NO-cGMP pathway play a role in the mechanisms of benzodiazepine withdrawal sensitivity (79).

Previous research has demonstrated increases in all three NOS isoforms at different stages of epileptogenesis. In an experimental epilepsy model, eNOS levels were found to increase within 3 to 24 h following the intracranial injection of kainate (80). However, iNOS and nNOS have been reported to be upregulated in a mouse model of electrically-induced epilepsy (81). In another study, in a rat model of epilepsy, the levels of nNOS and iNOS increased for 3 days following a seizure, and the increase persisted for a long period of time (82).

## 6. NOS inhibitors in the treatment of epilepsy

While numerous studies have demonstrated that NO and oxidative stress play a crucial role in the development of epilepsy, the anticonvulsant role of NOS inhibitors remains controversial. Some studies emphasize their anticonvulsant effects, while others highlight their proconvulsant effects (83). However, the efficacy of NOS inhibitors varies depending on the strength of their inhibition and isoform selectivity (76). Under physiological conditions, NO promotes the expression of activity-dependent neuroprotective proteins, whereas N-nitro-L-arginine methyl ester (L-NAME), a NOS inhibitor, or a soluble guanylate cyclase inhibitor, accelerates seizures in an epilepsy model. This suggests a crucial role for the NO-cGMP signaling pathway in epileptic seizures (16). Recent evidence has revealed a significant increase in 3-nitrotyrosine levels in iNOS-non-reactive microglia cells long following seizure induction (~3 months) (84). However, recent studies have shown that blocking NO production reduces epileptic seizures. However, the use of appropriate NOS inhibitors and the timing of treatment are crucial for a successful anticonvulsant effect (66,85).

L-NAME is a non-selective inhibitor of NOS enzymes, widely used in biochemical and pharmacological research. Side effects associated with NOS inhibition can be reduced by using a selective NOS inhibitor. Significant increases in nNOS expression can be observed in the early stages of epileptogenesis (69). nNOS is isolated in the postsynaptic membranes of neurons in interaction with the NMDA receptor-PSD-95 complex (68). NMDAR activation leads to increased calcium levels, resulting in nNOS activation, stimulating NO synthesis in neurons (Fig. 1). The increased expression of NO contributes to oxidative stress, ultimately leading to neurodegeneration and acute seizures. Nw-propyl-L-arginine (L-NPA), a specific inhibitor of nNOS, has a much higher affinity for iNOS and eNOS (69,86,87).

Evidence has indicated that L-NPA exerts a potent anticonvulsant effect in kainic acid-induced seizures. The administration of L-NPA has been shown to significantly reduce seizures and to result in a substantial decrease in epileptogenesis biomarkers measured at intervals (69). In another study, PTZ preconditioning was shown to exert an anticonvulsant effect against lithium-pilocarpine-induced status epilepticus in rats; however, this anticonvulsant effect was reversed by NOS inhibitors [L-NAME and 7-nitroindazole (7-NI)] (88). Furthermore, another study demonstrated that reductions in seizure-like events were observed following the administration of the nNOS selective inhibitor, 7-NI (89). In addition, the intraperitoneal administration of L-NAME (10 mg/kg) and 7-NI (30 mg/kg, i.p.) to mice was previously shown to enhance the anticonvulsant effect of ivermectin (90). In a PTZ kindling model of epilepsy, nNOS was shown to trigger hippocampal endoplasmic reticulum stress and oxidative damage via peroxynitrite (91). In the PTZ model, pentoxifyllin increased the seizure threshold, and this effect was potentiated by L-arginine. However, the administration of L-NAME and 7-nitroindazole resulted in a decrease in this effect (92). Genetic deletion of the nNOS gene significantly increases hippocampal neuropeptide Y expression, reducing hippocampal neuronal damage and cognitive decline caused by temporal lobe epilepsy (9). The co-administration of modafinil, a substance with alertness-enhancing and neuroprotective effects, with an NOS inhibitor has been shown to exert anticonvulsant effects in epileptic animals (93). Transcranial direct current stimulation in rats has been shown to suppress seizures by reducing TNF-α and IL-1β levels, and nNOS expression (94). Coenzyme Q10 has been reported to induce absence seizures in WAG/Rij rats by increasing NO levels (95). Studies on the effects of NOS inhibitors on epileptic seizures are presented in Table I (96-104).

Another NOS inhibitor with potential to reduce NO synthesis in the brain is iNOS inhibitor. Studies have shown that iNOS inhibitors have minimal effects on pain. However, positive results have been obtained when used in neurodegenerative diseases, cancer and experimental models of epilepsy (85). The most selective and potent iNOS inhibitor is N-(3-(Aminomethyl)benzyl)acetamidine (1,400 W) (105). 1400W is a nitric oxide inhibitor that is 5,000-fold more selective for iNOS compared to eNOS (106). In a previous *in vitro* study, a significant reduction in kainic acid-induced epileptiform spikes was shown following treatment with 1,400 W (64). In another study using animals, early epileptogenic neuronal hyperexcitability, glial cell proliferation, and neurodegeneration were significantly reduced after 1,400 W of kainat administration (64). The levels of pro-inflammatory mediators increase abruptly following brain injury, and these high levels persist for several months (66,84). The increase in 3-NT protein and iNOS activity, an indicator of nitro-oxidative stress in M1 type microglia cells in the brain, was significantly reduced with 1,400 W 1 week following the administration of kainat (64). Decreased iNOS activity in microglial cells is a possible consequence of the reduction in epileptic seizures and neurodegeneration. The results of these studies suggest that the neuroprotective effects of 1,400 W may occur by blocking the microglial supply of pro-inflammatory mediator molecules.

Despite the compelling anticonvulsant results obtained, significant obstacles remain as regards the clinical use of NOS inhibitors. The success observed in acute seizure models may not be equally consistent in chronic epilepsy. Furthermore, the blood-brain barrier crossing rates, systemic side-effects and therapeutic dose ranges of NOS inhibitors have not yet been fully optimized.

## 7. Conclusion and future perspectives

The present review discussed the complex effects of the NO signaling pathway on epileptogenesis, and seizure mechanisms and comprehensively reveals the anticonvulsant potential of NOS inhibitors. There is ample evidence to indicate that NO may play a bidirectional role (both neuroprotective and neurotoxic) in the central nervous system, demonstrating that specific NOS inhibition is a potent therapeutic strategy for raising the seizure threshold. The selective blockade of non-structural nNOS and iNOS isoforms suppresses glutamatergic hyperexcitability. nNOS inhibitors reduce neuronal damage by limiting intracellular Ca^2+^ influx and associated free radical production caused by NMDA receptor activation. By contrast, eNOS inhibition carries the risk of worsening seizures by disrupting cerebral blood flow. Therefore, ‘isoform specificity’ is crucial for the successful treatment of epilepsy. Future research is required to focus on developing next-generation smart molecules targeting nNOS and iNOS to improve the safety and efficacy of therapies targeting the NO pathway. From a real-world clinical perspective, the development of isoform-specific NOS inhibitors holds significant potential for pharmacoresistant epilepsy patients. Unlike broad-spectrum drugs, which often cause serious neurocognitive side-effects, these targeted therapies may provide a dual benefit: They can precisely suppress abnormal seizure activity while preserving the physiological NO pathways necessary for normal cognitive function.

## Figures and Tables

**Figure 1:**
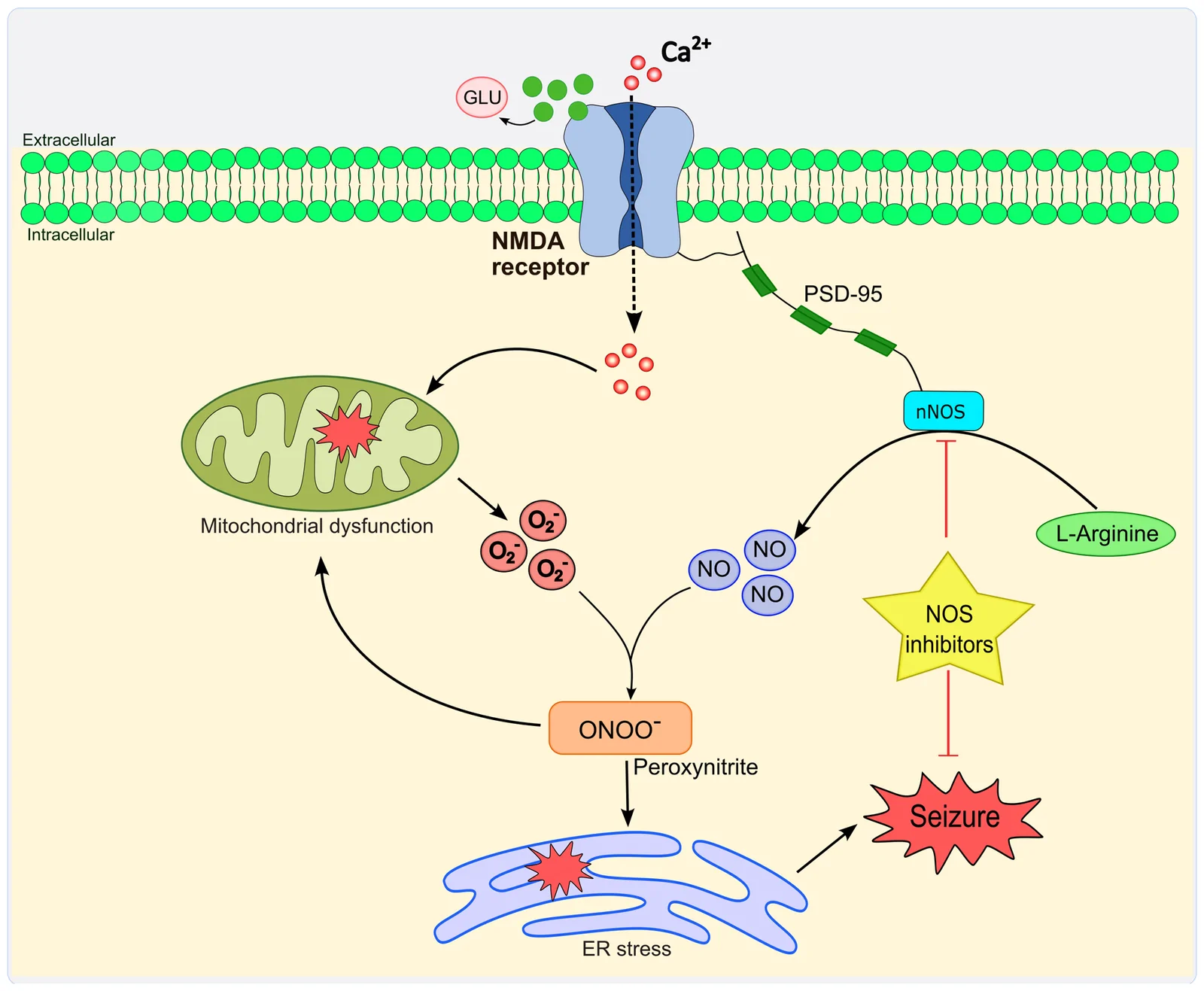
Schematic representation of the signaling pathway by which nNOS triggers hippocampal ER stress and oxidative damage via peroxynitrite in epileptic conditions, and the anticonvulsant mechanism of action of NOS inhibitors. During a seizure, it stimulates the NMDA receptor, which leads to nNOS activation and mitochondrial superoxide anion production. NO derived from nNOS rapidly reacts with the superoxide anion, producing large amounts of peroxynitrite. Peroxynitrite is neurotoxic, disrupts redox homeostasis, damages mitochondria, and causes ER stress in the hippocampus. All this leads to epilepsy. ER stress, endoplasmic reticulum stress; NOS, nitric oxide synthase; nNOS, neuronal nitric oxide synthase; NO, nitric oxide; NMDA, N-methyl-D-aspartate; ONOO^-^, peroxynitrite.

**Table I:** Proconvulsant/anticonvulsant activities of NOS inhibitors.

Compound/expression	Epilepsy model	Proconvulsant/anticonvulsant	NO/NOS inhibitors	(Refs.)
Genetic deletion of nNOS gene	Pilocarpine-induced TLE mice	Anticonvulsant	nNOS inhibition	(9)
Betulin	PTZ-induced seizure model	Anticonvulsant	Suppress iNOS/nNOS gene expressions	(12)
None	Rat model of genetic epilepsy	Anticonvulsant	Inhibition of nNOS by 7-NI	(14)
Allopurinol	PTZ and MES-induced seizure model	Anticonvulsant	Pretreatment with L-NAME, aminoguanidine, and 7-NI is a proconvulsant.	(73)
1400W (selective iNOS inhibitor)	DFP- and GD-induced SE	Anticonvulsant	nNOS inhibition	(85)
PTZ preconditioning	Lithium-pilocarpine-induced SE	Anticonvulsant	Pretreatment of L-NAME and 7-NI	(88)
nNOS knock-out mice	Seizure-like events (SLEs)	Anticonvulsant	nNOS inhibition	(89)
Ivermectin	PTZ model	Anticonvulsant	L-NAME (10 mg/kg) and 7-NI (30 mg/kg)	(90)
Genetic deletion of nNOS gene	PTZ model	Anticonvulsant	nNOS inhibition	(91)
Pentoxifyllin	PTZ model	Anticonvulsant Proconvulsant	L-arginine pretreatment L-NAME ve 7-NI pretreatment	(92)
Modafinil	Lithium-pilocarpine-induced SE	Anticonvulsant	Pretreatment of L-NAME (10 mg/kg), 7-NI (30 mg/kg), and aminoguanidine (100 mg/kg)	(93)
5-HT7 receptor antagonist SB269970	PTZ model	Anticonvulsant	7-NI inhibition of nNOS	(96)
Nos1 conditional knockout mice	Pilocarpine-induced TLE mice	Proconvulsant	nNOS inhibition	(97)
Fingolimod	PTZ kindling	Anticonvulsant	The L-NAME reduced the P-gp expression	(98)
Aminoguanidine	PTZ model; pilocarpine-induced SE	Anticonvulsant	iNOS inhibition	(99)
Valproate (300 mg/kg)	PTZ kindled animals	Anticonvulsant	Inhibition of nNOS by 7-NI	(100)
Morphine	Lithium-pilocarpine-induced SE	Anti-convulsant	Pretreatment with L-NAME, 7-NI, and aminoguanidine reversed the inhibitory effect of morphine.	(101)
WIN (cannabinoid agonist)	Pilocarpine-induced acute seizures	Anticonvulsant	Pretreatment with 7NI enhances anticonvulsant action of WIN	(102)
None	PTZ-induced convulsive seizure	Anticonvulsant	L-NNA, 7-NI	(103)
Nanocurcumin	PTZ-induced seizure	Anticonvulsant	Pretreatment with L-NAME	(104)

NOS, nitric oxide synthase; NO, nitric oxide; nNOS, neuronal nitric oxide synthase; SE, status epilepticus; PTZ, pentylenetetrazole; 7-NI, 7-nitroindazole; DFP, diisopropylfluorophosphate; GD, soman; MES, maximal electroshock; 7-NI, 7-nitroindazole; L-NAME, N-nitro-L-arginine methyl ester.

## Data Availability

Not applicable.
